# AIding, Not Replacing, Clinicians: A Machine-Based Learning Approach to Predict Successful Outcomes in Transcatheter Tricuspid Valve Interventions

**DOI:** 10.1016/j.jacadv.2024.101574

**Published:** 2025-01-21

**Authors:** Sean Lal

**Affiliations:** a Department of Cardiology, Royal Prince Alfred Hospital, Sydney, Australia; b Faculty of Medicine and Health, The University of Sydney, Sydney, Australia

**Keywords:** computer modeling, transcatheter interventions, tricuspid regurgitation

**Figure:**
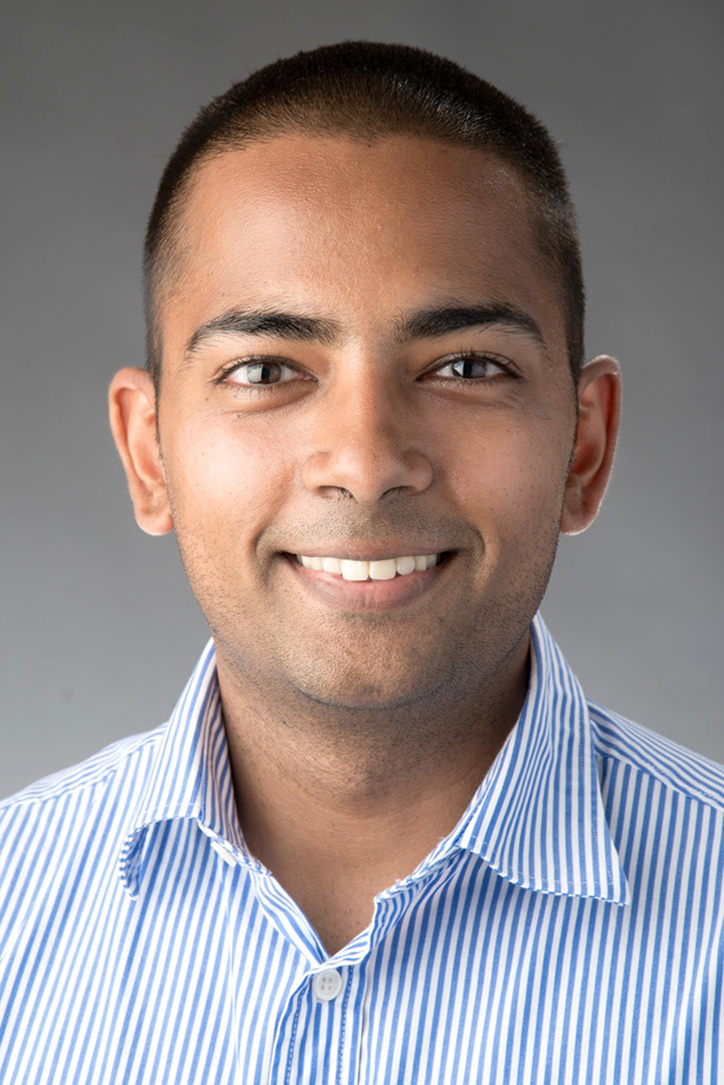

The tricuspid valve (TV) is a complex piece of anatomy. There can be variation in the number and size of the anterior, posterior, and septal leaflets with analogous variation in the subvalvular apparatus of chordae and papillary muscles.^1^^,^^2^ Notably, the septal leaflet directly attaches to the ventricular septum, distinguishing it from the other heart valves. Then, there is the TV annulus, which is nonplanar and more oval-shaped than the saddle-shaped mitral annulus. It is also highly dynamic, adjusting its shape during the cardiac cycle and in response to loading conditions. Normally, the TV annulus has an elliptical structure, but in patients with functional tricuspid regurgitation (TR), it can become planar and circular due to annular dilation, which primarily occurs in the anteroposterior plane due to its medial attachments to the atrial-ventricular fibro skeleton.^3^ Therefore, percutaneous interventions to the TV are relatively more complex than for the mitral valve but we are now entering a primetime era.

Advances in our understanding of TV anatomy and how it links to the heart failure axis (left ventricular dysfunction – left atrial myopathy – pulmonary hypertension – right atrial myopathy) means that TR is no longer underappreciated. It is recognised as an independent predictor of morbidity and mortality.^1^^,^^4^^,^^5^ Fixing the problem is becoming easier, given the modern advances in percutaneous options to intervene on heart valves. However, not dissimilar to historically poor outcomes in patients undergoing isolated TR surgery (there was a bias to these patients being sicker), there remains the question as to how we can best determine who will benefit from TV interventions.^1^^,^^4^

In this issue of *JACC: Advances*, Fortmeier et al. presents a “simplified outcome prediction in patients undergoing transcatheter tricuspid valve intervention by survival tree-based modelling”.^6^ The authors introduce a novel machine learning-based model for predicting survival outcomes in patients undergoing transcatheter tricuspid valve intervention (TTVI) for severe TR. This model was derived from data from 918 patients, including preprocedural clinical, biochemical, echocardiographic, and hemodynamic data. The aim was to create a simple tool to stratify patients based on their risk of mortality after TTVI.

Using supervised machine learning, Fortmeier et al. identified 4 key clinical predictors of mortality: mean pulmonary artery pressure (mPAP), NT-proBNP levels, right atrial (RA) area, and estimated glomerular filtration rate (eGFR). These parameters were used to create a survival tree-based model, which classified patients into 2 risk categories: low, intermediate, and high. The low-risk group (mPAP ≤28 mm Hg and NT-proBNP ≤2,728 pg/mL) had a 2-year survival rate of 85.5%, while the high-risk group (mPAP >28 mm Hg, RA area >32.5 cm^2^, and eGFR ≤51 mL/min) had a significantly worse 2-year survival of only 52.6%.

The performance of this model was then compared to established risk scores such as the TRI-Score and EuroScore II and it was shown to be relatively superior in stratifying high-risk patients. This highlights a key strength of the study in the external validation of the model, which demonstrates reliability in predicting outcomes in specific clinical settings.

The simplicity of the model makes it an attractive tool for personalized clinical practice when compared to more complex scoring systems. However, the utility of such models should be to inform rather than replace a heart team-based approach regarding treatment decisions, optimizing patient selection for TTVI, and hence identifying high-risk individuals.

There are also several limitations in the study that are worth noting. The absence of a randomized control group of patients receiving conservative treatment means one cannot demonstrate an advantage over noninterventional care, which is true for all TTVI intervention studies in determining impact on overall survival.^7^ There is also the retrospective design of this study, which introduces potential selections biases.

It should be noted that echocardiographic estimates for pulmonary hypertension can be discrepant, particularly with severe TR, when compared to gold standard of right heart catheterization.^8^ The methodology for the artificial intelligence-derived prediction of pulmonary artery (PA) pressures by Fortmeier et al. relied on several input variables including left ventricular ejection fraction, left ventricular end-systolic diameter, left atrial area, estimated systolic pulmonary artery pressure (sPAP), basal right ventricular diameter, tricuspid annular plane systolic excursion (TAPSE), TR vena contracta width, tricuspid valve effective regurgitant orifice area, right atrial (RA) area, and inferior vena cava diameter. But we know, for example, that right ventricular (RV) strain and magnetic resonance imaging–based measurements of the RV are superior for assessing right heart remodeling, and hence incorporating such advanced imaging techniques in clinical decision making could improve risk stratification in patients with complex RV dysfunction.^9^

There is also the importance of RV-PA coupling in relation to RV contractile reserve, which is paramount when the relative “pressure release” for the RV is removed when severe TR is corrected. Use of TAPSE and sPAP measures to assess the RV-PA coupling in patients with moderate-severe TR improves risk prediction compared to isolated echocardiographic measures of RV function,^10^ but it should be noted that TAPSE represents only the longitudinal shortening of the RV free-wall. Given that the inlet and outlet of the RV are at right-angles to each other, there is a need to account for the effective forward flow from the RV to the pulmonary vasculature, which is not fully captured when examining the RV free wall alone.

With the ageing population of the world, coupled with increasing rates of obesity and diabetes, we need to consider the increasing proportion of heart failure related TR that is ultimately the result of heart failure with preserved ejection fraction. The 4 key predictors of mortality (mPAP, NT-proBNP levels, RA area, and eGFR) identified by Fortmeier et al. in their model are also important considerations in heart failure with preserved ejection fraction associated morbidity and mortality.^11^^,^^12^ Fixing TR in patients with heart failure with preserved ejection fraction may not necessarily improve their symptoms or their future risk of heart failure admissions, and so we need to broaden our considerations and applicability of such machine-based learning models to incorporate diverse clinical populations that have the commonality of moderate-severe TR.

In conclusion, Fortmeier et al. provides a significant advancement in our field regarding the capabilities of machine-based learning tools to predict patient risk. Specifically, their model for outcome prediction in patients undergoing TTVI has the potential to be an important (but not the only) tool for specialized heart teams to aid in clinical decision-making for individual patients.

## Funding support and author disclosures

The author has reported that he have no relationships relevant to the contents of this paper to disclose.
